# Regulatory effects of Nr4a2 on Th2 cells from patients with pemphigus vulgaris

**DOI:** 10.18632/oncotarget.24371

**Published:** 2018-02-01

**Authors:** Jianbo Chen, Yao Zhang, Yunsheng Liang, Ming Zhao, Hai Long, Rong Xiao, Haijing Wu, Jieyue Liao, Shuaihantian Luo, Guiying Zhang, Qianjin Lu

**Affiliations:** ^1^ Department of Dermatology, Hunan Key Laboratory of Medical Epigenomics, The Second Xiangya Hospital of Central South University, Changsha 410011, China

**Keywords:** cytokines, negative regulation, Nr4a2, pemphigus vulgaris, Th2 cells

## Abstract

Pemphigus vulgaris is an autoimmune blistering disease characterized by a loss of epidermal cell–cell adhesion caused by anti-desmoglein (Dsg) autoantibodies. The pathogenesis of PV remains unclear. However, abnormal frequency and function of Th2 cells are believed to contribute to PV. We investigated Nr4a2, a transcription factor, which has been found to regulate T cell differentiation, for its association with Th2 cell differentiation and functions in PV. We found significantly decreased mRNA and protein levels of Nr4a2 in CD4+ T cells from patients with PV, compared with healthy control subjects. In addition, mRNA and protein levels of Nr4a2 in CD4+ T cells were inversely correlated with serum levels of IL-4 and IL-13 in patients with PV. Overexpression of Nr4a2 in CD4+ T cells from patients with PV significantly reduced the mRNA levels of GATA3, IL-4, and IL-13, while Nr4a2 siRNA treatment showed the reverse effects on the expression of these Th2-related cytokines and transcription factors. The data suggest that the altered level of Nr4a2 in CD4+ T cells is associated with the development of PV. Nr4a2 may contribute to the pathogenesis of PV by negatively regulating Th2 activity and secretion of Th2-related cytokines.

## INTRODUCTION

Pemphigus vulgaris (PV) is an autoimmune blistering disease, caused by anti-desmoglein (Dsg) immunoglobulin G (IgG) autoantibodies. Desmogleins are regarded as the principal auto-antigens in pemphigus. The relevant protease, generated by auto-reactive antibodies through cell transduction pathways, results in a loss of epidermal cell–cell adhesion, leading to the occurrence of PV [1, 2].

The pathogenesis and molecular mechanisms underlying PV are sophisticated and not well elucidated. T helper (Th) cells, also known as CD4+ T cells, play important roles in the pathogenesis of PV [3, 4]. Different subsets of Th cells exert different effects and form a complicated network to orchestrate the autoimmune inflammation. Previous studies have revealed that PV is a Th2-dominant disease [5, 6]. Direct evidence of this is that Dsg3-specific Th2 activity positively correlates with the titer of anti-Dsg3 antibody [7]. The transcription factor GATA-3 is regarded as a master regulator of Th2 cell differentiation, during which it upregulates the expression of Th2-type cytokines interleukin-4 (IL-4), IL-5, and IL-13 [8–10]. IL-4, a representative Th2 cytokine, promotes proliferation of T cells, enhances antibody production by B cells, and is involved in the development of Th2-dominant autoimmune disease [10, 11]. IL-13 has a 30% protein homology with IL-4 and shares IL-4Rα as the receptor in common with IL-4; it is thus presumed to have an identical effector function to IL-4 [12, 13]. In addition, regulatory T cells (Tregs), another Th cell subset with a negative regulatory effect, modulate the differentiation of Th cells, especially Th2 cells, and are considered to hinder the development of PV [2, 14]. The imbalance between abnormal activation of Th2 cells and reduced Treg cell activity induces disorder of the immune system and activates B cells to produce specific autoantibodies contributing to pemphigus.

Nr4a2, a transcription factor and a member of the nuclear hormone receptor (Nr4a) family, combines with related response elements to regulate gene transcription. Recent research demonstrates that Nr4a2 participates in the differentiation of CD4+ T cells and plays an important role in autoimmune diseases [15, 16]. Saijo *et al.* [17] have confirmed that Nr4a2 is a transcription factor with bidirectional regulation effects. Sekiya *et al.* have found that Nr4a2 combines with the regulatory regions of Foxp3 and strongly induces Foxp3 in naturally occurring Tregs (nTregs) by mediating active histone modification, consequently regulating the differentiation and maturation of Tregs [18, 19]. Furthermore, knocking out Nr4a2 in mice can trigger an abnormal activation of Th2 cells and a Th2-type autoimmune response, leading to the development of systemic multiorgan autoimmune diseases. Together, these findings identify Nr4a2 as a fundamental component in the regulation of T helper cell differentiation and development, namely, a positive regulation of Tregs and a negative regulation of Th2 cells, demonstrating its crucial roles in autoimmune diseases [20].

Therefore, we hypothesized that the Th2 cells are upregulated in PV, owing to the downregulated Nr4a2, which facilitates the activation of the humoral immune machinery and triggers autoimmunity in pemphigus. In this study, we compared Nr4a2 expression in CD4+ T cells from patients with PV and from healthy control subjects. The expression levels of Th2-related cytokines IL-4 and IL-13, as well as the key transcription factor of Th2 cells, GATA3, in CD4+ T cells were compared between subjects with PV and healthy control subjects. The correlation between Nr4a2 expression and cytokine levels of IL-4 and IL-13 was also analyzed. Further, we transfected CD4+ T cells *in vitro* with Nr4a2-overexpressing plasmid and Nr4a2-siRNA, respectively, and thus examined the regulatory mechanisms of Nr4a2 on Th2 cells in the settings of PV.

## RESULTS

### Nr4a2 expression is significantly decreased in CD4+ T cells from patients with PV

**Figure 1:**
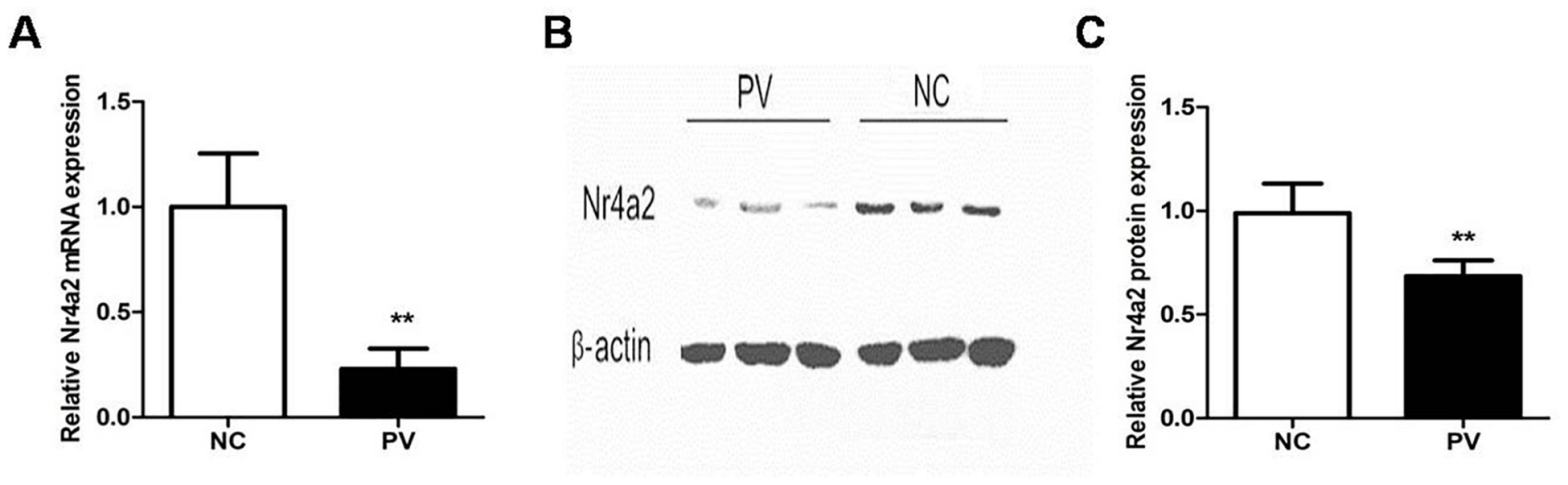
Expression of Nr4a2 in CD4+ T cells from patients with PV and healthy control subjects **(A)** Relative expression level of Nr4a2 mRNA in CD4+ T cells measured by RT-PCR and normalized to β-actin. The Nr4a2 mRNA level was significantly decreased in patients with PV compared with healthy control subjects (*p* = 0.006). **(B)** Representative Western blot of Nr4a2 and β-actin expression in CD4+ T cells from patients with PV and healthy control subjects. **(C)** Quantitative analysis of band intensities of Nr4a2 normalized to β-actin. The Nr4a2 protein level in CD4+ T cells was decreased in patients with PV (*p* = 0.007). (^*^p < 0.05, ^**^p < 0.01, ^***^p < 0.001).

To detect the expression of Nr4a2 in CD4+ T cells from patients with PV, we isolated RNA and used real-time PCR to measure and compare Nr4a2 mRNA levels. Compared with healthy control subjects, the expression level of Nr4a2 relative to β-actin was significantly decreased in patients with PV (0.230 ± 0.096 vs. 1.000 ± 0.254, *p* = 0.006; Figure 1A). To confirm the expression level of Nr4a2 in patients with PV, we performed Western blotting with the proteins extracted from CD4+ T cells. The protein level of Nr4a2 was also decreased in CD4+ T cells from patients with PV, compared with those from healthy control subjects (0.727 ± 0.055 vs. 1.000 ± 0.072, *p* = 0.007; Figure 1B and 1C).

### Expression of GATA3, IL-4, and IL-13 is increased in CD4+ T cells from patients with PV

**Figure 2:**
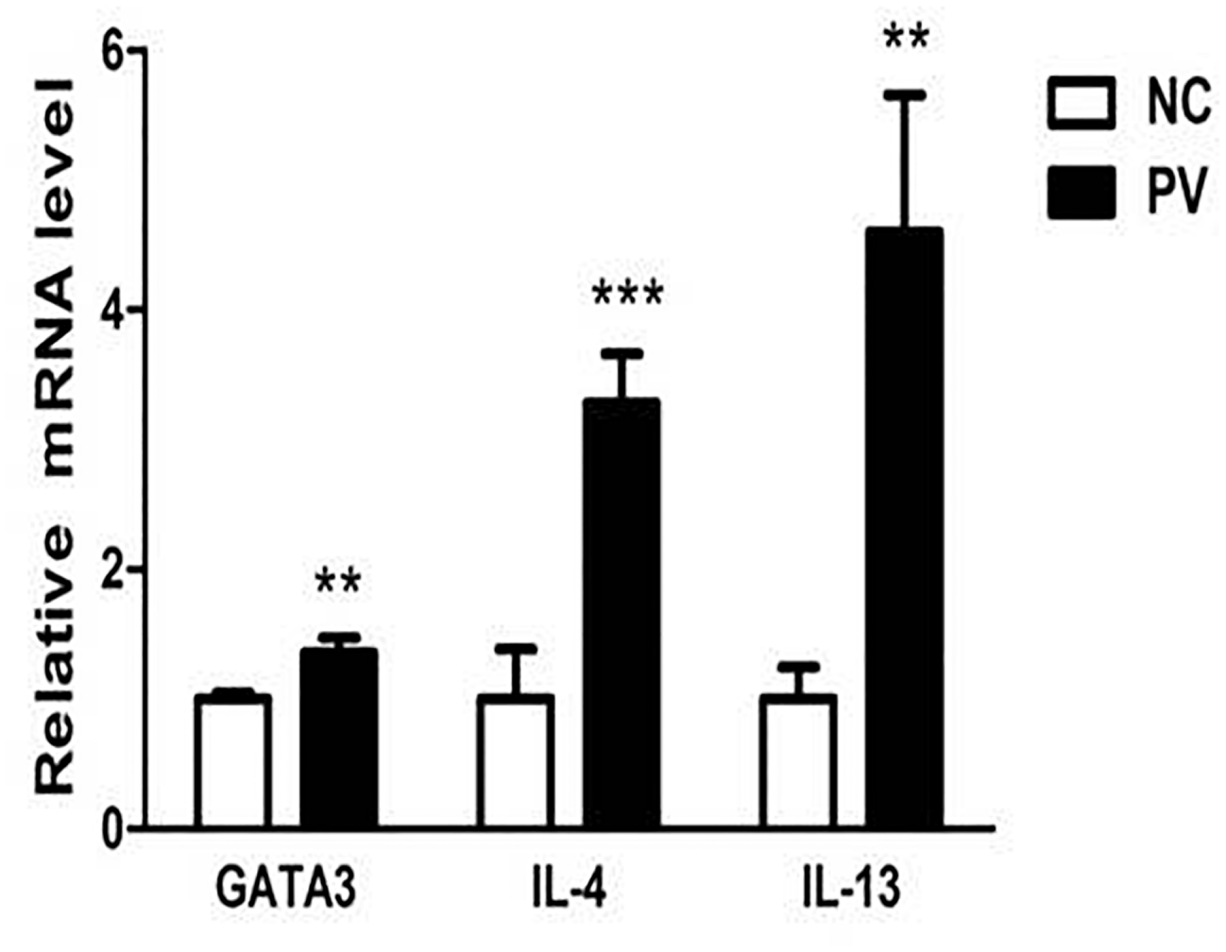
Relative expression of GATA3, IL-4, and IL-13 mRNA in CD4+ T cells from PV patients, measured by real-time quantitative RT-PCR and normalized to β-actin (*p* = 0.001, *p* < 0.001, *p* = 0.008, respectively) (^*^p < 0.05, ^**^p < 0.01, ^***^p < 0.001).

Real-time PCR was also used to detect the mRNA levels of GATA3, IL-4, and IL-13 in CD4+ T cells from 19 patients with PV and 19 healthy control subjects. Through this quantitative experiment, we demonstrated that the mRNA levels of GATA3, IL-4, and IL-13 are elevated in CD4+ T cells from patients with PV, compared with those from healthy control subjects (1.359 ± 0.113 vs. 1.000 ± 0.048, *p* = 0.001; 3.282 ± 0.383 vs. 1.000 ± 0.388, *p* < 0.001; 4.602 ± 1.053 vs. 1.000 ± 0.249, *p* = 0.008, respectively; Figure 2).

In addition, we collected corresponding serum samples from the experimental subjects, and determined the serum concentration of IL-4 and IL-13 by ELISA. As displayed in Figure 4A and 4B, serum levels of IL-4 and IL-13 were also increased in patients with PV, compared with healthy control subjects (28.65 ± 0.803 pg/ml vs. 24.15 ± 1.059 pg/ml, *p* = 0.001; 43.67 ± 3.624 pg/ml vs. 31.44 ± 2.746 pg/ml, *p* = 0.013; respectively). These results were in accord with the increased mRNA expression levels of IL-4 and IL-13 in CD4+ T cells from patients with PV.

**Figure 3:**
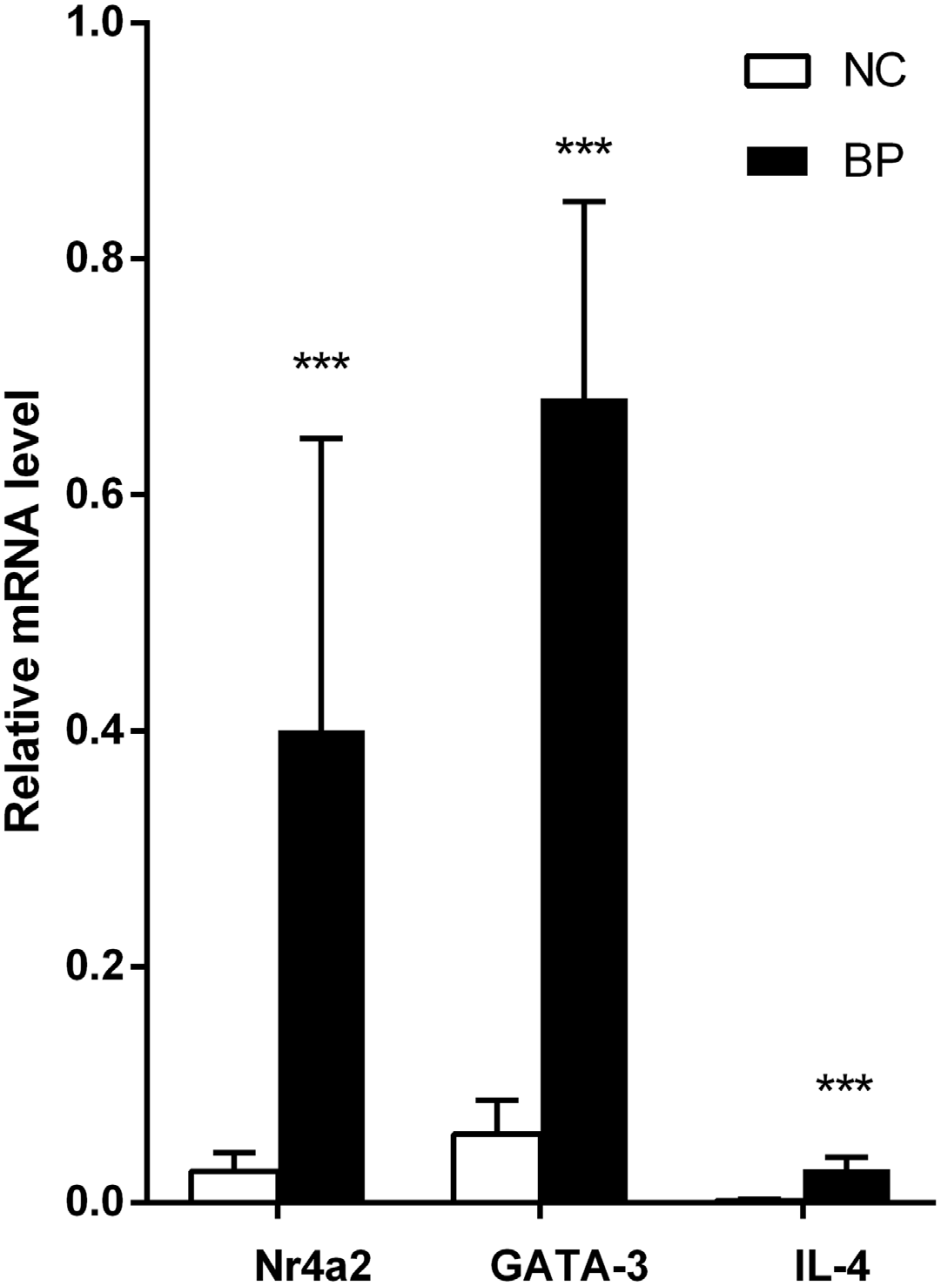
Relative expression of GATA3, nr4a2, and IL-4 mRNA in CD4+ T cells from BP patients, measured by real-time quantitative RT-PCR and normalized to β-actin (*p* <0.001, *p* < 0.001, *p* < 0.001, respectively) (^*^p < 0.05, ^**^p < 0.01, ^***^p < 0.001).

**Figure 4:**
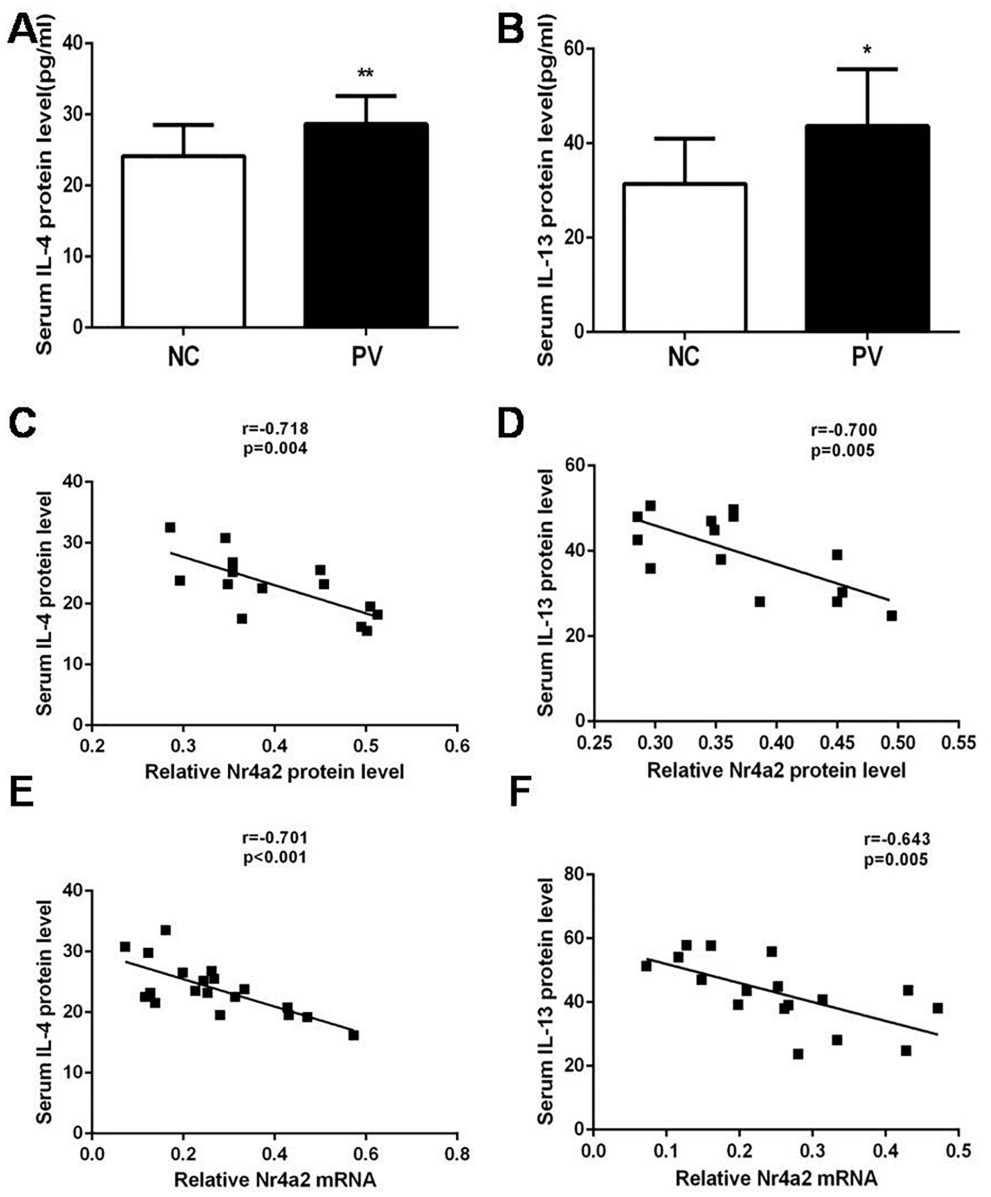
Serum levels of **(A)** IL-4 and **(B)** IL-13 in patients with PV (*n* = 19) and healthy control subjects (*n* = 19), as detected by ELISA. In patients with PV, Nr4a2 expression correlated negatively with corresponding serum levels of **(C)** IL-4 and **(D)** IL-13. The Nr4a2 mRNA level was also negatively correlated with serum levels of **(E)** IL-4 and **(F)** IL-13. (^*^p < 0.05, ^**^p < 0.01, ^***^p < 0.001).

### Expression of Nr4a2, GATA3 and IL-4 is increased in CD4+ T cells from patients with BP

Real-time PCR was also used to detect the mRNA levels of Nr4a2 GATA3, and IL-4 in CD4+ T cells from 6 patients with BP and 7 healthy control subjects. Through this quantitative experiment, we demonstrated that the mRNA levels of Nr4a2, GATA3, and IL-4 are elevated in CD4+ T cells from patients with BP, compared with those from healthy control subjects (0.400 ± 0.194 vs. 0.027 ± 0.012, *p* < 0.001; 0.682 ± 0.130 vs. 0.058 ± 0.023, *p* < 0.001; 0.029 ± 0.007 vs. 0.002 ± 0.001, *p* < 0.001, respectively; Figure 3).

### An inverse correlation between Nr4a2 expression in CD4+ T cells and the serum level of IL4 and IL-13 in patients with PV

We subsequently analyzed the relationship between the expression of Nr4a2 in CD4+ T cells and the serum level of the cytokines IL-4 and IL-13 in patients with PV [Figure 4A and 4B]. As shown in Figure 4C and 4D, there was a significant inverse correlation between the protein expression level of Nr4a2 in CD4+ T cells and the serum level of both IL-4 and IL-13 (*r* = −0.718, *p* = 0.004; *r* = −0.700, *p* = 0.005). There was a similar inverse correlation between the relative mRNA expression level of Nr4a2 in CD4+ T cells and the serum level of both IL4 and IL-13(*r* = −0.701, *p* < 0.001; *r* = −0.643, *p* = 0.005; Figure 4E and 4F).

### Overexpression of Nr4a2 suppressed expression of GATA3 and Th2-related cytokines in CD4+ T cells from PV patients

**Figure 5:**
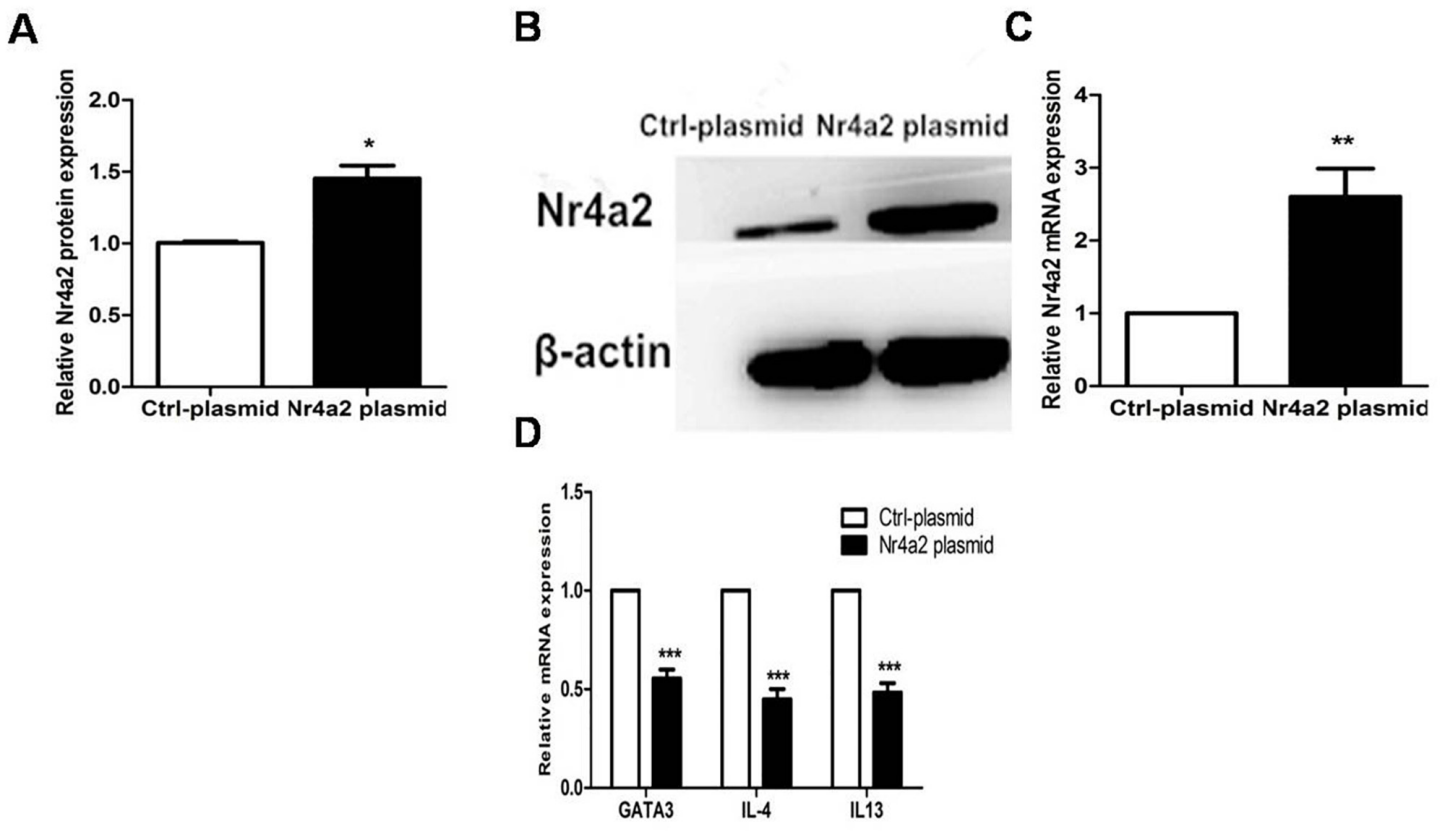
**(A–C)** Protein and mRNA expressions of Nr4a2 were significantly elevated after transfection with Nr4a2-overexpressing plasmid in CD4+ T cells from patients with PV (*p* = 0.0192, *p* = 0.001, respectively). **(D)** mRNA expression levels of GATA3, IL-4, and IL-13 were significantly decreased after transfection with Nr4a2-overexpressing plasmid in CD4+ T cells from patients with PV (*p* < 0.001; *p* < 0.001, *p* < 0.001, respectively). (^*^p < 0.05, ^**^p < 0.01, ^***^p < 0.001).

We constructed an Nr4a2-overexpressing plasmid and a vehicle plasmid as a control, and transfected these plasmids, respectively, into CD4+ T cells from PV patients. Compared with the vehicle controls, the protein and mRNA expression levels of Nr4a2 were significantly elevated after transfection with Nr4a2-overexpressing plasmid into the CD4+ T cells (1.453 ± 0.179 vs. 1.000 ± 0.018, *p* = 0.0192; 2.602 ± 0.775 vs. 1.000 ± 0.000, *p* = 0.001, respectively; Figure 5A–5C), and downregulated the mRNA expression level of GATA3, IL-4, and IL-13 in CD4+ T cells from patients with PV, compared with the vehicle controls (0.554 ± 0.090 vs. 1.000 ± 0.000, *p* < 0.001; 0.448 ± 0.105 vs. 1.000 ± 0.000, *p* < 0.001; 0.483 ± 0.097 vs. 1.000 ± 0.000, *p* < 0.001; Figure 5D).

### Targeting Nr4a2 by siRNA upregulated the expression of GATA3 and Th2-related cytokines in CD4+ T cells from healthy donors

**Figure 6:**
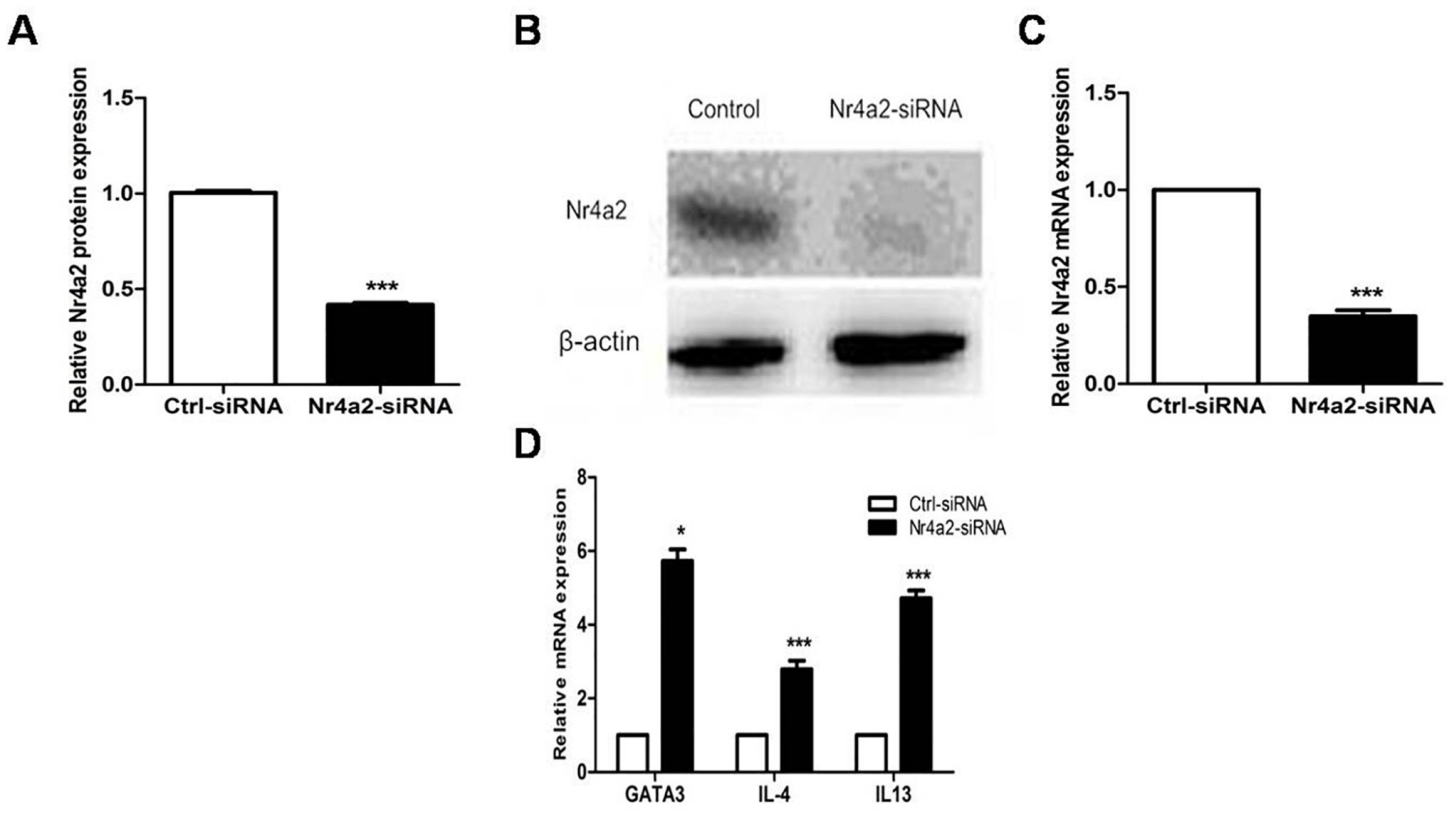
**(A–C)** Protein and mRNA expression levels of Nr4a2 were significantly decreased after treatment with Nr4a2-siRNA in CD4+ T cells from healthy control subjects (*p* < 0.001, *p* < 0.001, respectively). **(D)** mRNA expression levels of GATA3, IL-4, and IL-13 were significantly elevated after treatment with Nr4a2-siRNA in CD4+ T cells from healthy control subjects (*p* = 0.01, *p* < 0.001, *p* < 0.001, respectively). (^*^p < 0.05, ^**^p < 0.01, ^***^p < 0.001).

We transfected CD4+ T cells from healthy donors using an Nr4a2-siRNA and a control siRNA. Compared with the controls, the CD4+ T cells transfected with the Nr4a2-siRNA showed decreased protein and mRNA expressions of Nr4a2 (0.418 ± 0.025 vs. 1.000 ± 0.023, *p* < 0.001; 0.348 ± 0.075 vs. 1.000 ± 0.000, *p* < 0.001; Figure 6A–6C). Meanwhile, mRNA levels of GATA3, IL-4, and IL-13, examined by real-time PCR, were significantly elevated after transfection with the Nr4a2-siRNA in CD4+ T cells (5.731 ± 0.757 vs. 1.000 ± 0.000, *p* = 0.01; 2.793 ± 0.561 vs. 1.000 ± 0.000, *p* < 0.001; 4.718 ± 0.521 vs. 1.000 ± 0.000, *p* < 0.001; Figure 6D).

## DISCUSSION

The autoimmune response mediated by Th cells, especially the abnormal increase of Th2 cells and the decrease of Treg cells, plays pivotal roles in the pathogenesis of PV. It has been previously demonstrated that Nr4a2 positively regulates Tregs in autoimmune responses. Consistently, our experiments demonstrated that the expression level of Nr4a2 in CD4+ T cells from patients with PV were significantly lower than in those from healthy control subjects, suggesting a potentially important role of Nr4a2 in the pathogenesis of PV. However, how Nr4a2 participates in the pathogenesis of PV; especially, whether it is also involved in the regulation of Th2 immune response in PV, remains to be elucidated. Our data provide positive answers to these questions.

Our results showed increased expression of the Th2-type cytokines IL-4 and IL-13 in CD4+ T cells and elevated serum levels of these two cytokines in patients with PV. This was consistent with previous studies, highlighting the significance of the activation of Th2 response in the pathogenesis of PV. For instance, Caproni *et al.* [21] characterized the immunophenotype of the cellular infiltrate of pemphigus lesional skin and studied the secreted cytokines, including IL-4, IL-13, and IFN-γ. Caproni *et al.* [21] identified the presence of a T cell population with a prevalent Th2-like cytokine pattern in pemphigus lesional skin. Veldman *et al.* [2] demonstrated that the level of IL-4 was increased in the sera of patients with PV. Rizzo *et al.* evaluated IL-4-producing CD4+ T cells using peripheral blood mononuclear cells from patients with active or remittent PV and healthy control subjects, and found that the mean frequency of Th2 cells was significantly elevated in patients with PV with active disease [7]. Takahashi *et al.* [22] stimulated Dsg3^−^/^−^ mice with soluble recombinant CD40L and immunized them with recombinant Dsg3 (rDsg3) to obtain culture supernatants of splenic B cells, in the presence of exogenous IL-4, IL-10, or IFN-γ. The IgG anti-Dsg3 antibodies were only detected in the aforementioned supernatants with exogenous IL-4, but not in those with IL-10 or IFN-γ. Next, Takahashi *et al.* [22] administered soluble cytokine receptor IL-4R to Rag-2^−^/^−^ mice, which can neutralize IL-4 *in vivo*. Five days later, Takahashi *et al.* [22] adoptively transferred the pathogenic Dsg3-reactive T cell clone 147#48 and primed Dsg3^−^/^−^ B cells into the immuno-deficient mice. The IL-4R significantly suppressed the production of IgG anti-Dsg3 antibodies [23], indicating the critical role of IL-4 in promoting B cells to produce specific anti-Dsg3 antibodies and in inducing the occurrence of PV. Interestingly, bullous pemphigoid, another type of autoimmune blistering disease similar to, but distinct from, PV, is also featured by a Th2-predominant autoimmunity, showing significantly increased serum IL-4 and IL-13 as compared with healthy individuals [24].

Moreover, our data identified an increased expression of GATA3, a major regulator of Th2 cells, in CD4+ T cells from patients with PV. GATA3 can promote the conversion of Th0 cells to Th2 cells, and restrict the process of Th0 cells to Th1 cells [25, 26]. Because of the predominant expression in Th2 cells and its prominent function, GATA3 is an effective index to measure the functional status of Th2 cells. Therefore, abnormally high GATA3 expression is responsible for Th2-biased autoimmunity in PV [27]. However, the mRNA levels of Nr4a2, GATA3, and IL-4 are elevated in CD4+ T cells from patients with BP, compared with those from healthy control subjects. This result also shows that Nr4a2 is only specifically expressed in PV patients.

As shown by our results, the expression of Nr4a2 is inversely correlated with the serum levels of IL-4 and IL-13. This highlights the potential of Nr4a2 as a critical molecule that regulates Th2-type response in PV, which has been further investigated and confirmed by our studies using Nr4a2-overexpressing plasmid and Nr4a2 inhibitors. The significant influence of artificially modulated expression of Nr4a2 on Th2-related cytokines suggests that Nr4a2 can negatively regulate GATA3 and thus modulate the Th2-type cytokines IL-4 and IL-13. Reversing the reduced expression of Nr4a2 in patients with PV can restrain the activity of Th2-type immunity.

In summary, our findings indicate that aberrantly decreased expression of Nr4a2 may lead to an exacerbation of pathogenic Th2-type immunity and contribute to the pathogenesis of PV. The exact molecular mechanisms of this negative regulation of Th2-type immunity by Nr4a2 warrant further investigation. A possible hypothesis is that Nr4a2 may directly bind to the promoter region of GATA3, or to that of IL-4 or IL-13, and thus modulate its expression. Further study into these mechanisms may shed new light on our understanding of the pathogenesis of PV and would provide novel molecular targets for diagnosis and treatment of this disease.

## MATERIALS AND METHODS

### Subjects

Patients with PV (*n* = 25, mean age 49.8 ± 2.6 years) and patients with bullous pemphigoid (BP) (*n* = 6, mean age 53.6 ± 3.8 years) were recruited from the Outpatient Department of Dermatology and the Dermatology Ward at the Second Xiangya Hospital of Central South University. All patients fulfilled the Japanese diagnostic criteria for pemphigus vulgaris, and the severity index was assessed using previously published guidelines [28]. Demographic and relevant clinical information for the patients included in the study is presented in Table 1. Nineteen healthy control subjects (*n* = 19, mean age 47.5 ± 3.6 years) were recruited from medical staff at the Second Xiangya Hospital of Central South University. The study was approved by the ethics committee of Xiangya School of Medicine, the Central South University; written informed consent was obtained from all subjects.

**Table 1:** Patient demographics and medications

Patient	Sex/age	Revised severity index	Medications
1	F/64	severe	Pred 100mg/day
2	F/56	severe	Pred 75mg/day
3	M/40	severe	Pred 75mg/day
4	F/50	moderate	None
5	F/45	moderate	None
6	F/48	severe	Pred 50mg/day,MTX 15mg/week
7	F/48	severe	Pred 100mg/day,MMF 1.5g/day
8	M/59	moderate	Pred 30mg/day
9	F/40	moderate	None
10	F/40	severe	Pred 100mg/day, MTX 15mg/week
11	M/50	severe	Pred 100mg/day
12	M/62	moderate	Pred 40mg/day
13	M/49	severe	Pred 65mg/day
14	M/40	mild	None
15	M/36	moderate	None
16	M/48	moderate	Pred 40mg/day
17	M/62	severe	Pred 100mg/day
18	M/51	severe	None
19	F/45	severe	None

F: female; M: male; Pred: prednisone; MTX: methotrexate; MMF: mycophenolatemofetil.

### Isolation of peripheral blood mononuclear cells and CD4+ T cells

A sample (60 ml) of venous peripheral blood was withdrawn from each patient or control subject, and preserved in heparin. Peripheral blood mononuclear cells were isolated by Ficoll-Hypaque density gradient centrifugation (Shanghai Hengxin Chemical Reagent Co., Shanghai, China). CD4+ T cells were isolated by positive selection using magnetic CD4 affinity beads, according to the manufacturer’s protocol (MiltenyiBiotec, Bergisch Gladbach, Germany).

### RNA isolation and real-time quantitative PCR

Total RNA from CD4+ T cells was isolated using Rneasy mini kits (Qiagen, Venlo, the Netherlands), and mRNA levels were quantified by real-time PCR in a thermal cycler (LightCycle96, Roche, Switzerland), using a commercial kit (Quanti Tect SYBR Green Real-time PCR kit, Qiagen) and the primers listed in Table 2. In addition, β-actin was amplified as a loading control (Table 2). A dilution series of sample RNA was included to generate a standard curve, which was used to calculate relative concentrations of transcript in each RNA sample.

**Table 2:** Primer sequences used for real-time RT-PCR

Gene	Primer	Sequence 5’→3’
Nr4a2	Forward	CACTTCTCTCCCCAGCTTCA
	Reverse	GTGTTGCTGGTAGTTGTGCA
GATA3	Forward	AGATGGCACGGGACACTACC
	Reverse	GTTGTGGTGGTCTGACAGTTCG
IL-4	Forward	CGGCAACTTTGTCCACG
	Reverse	TCTGTTACGGTCAACTCGGT
IL-13	Forward	CCACGGTCATTGCTCTCACT
	Reverse	GCTCCATACCATGCTGCCAT
β-actin	Forward	CATGTACGTTGCTATCCAGGC
	Reverse	CTCCTTAATGTCACGCACGAT

### Western blotting

Whole-cell extracts were lysed by cell lysis in RIPA buffer supplemented with standard protease inhibitors, according to the manufacturer’s instructions (KeyGEN BioTECH, China). Proteins were separated in a 10% SDS-PAGE gel and were transferred to polyvinylidene difluoride (PVDF) membranes for immune blotting. The PVDF membranes were treated for 1 h with a blocking buffer, and then incubated with mouse anti-β-actin monoclonal antibodies (1:2000, Santa Cruz, CA, USA) or mouse anti-Nr4a2 monoclonal antibodies (1:1000, Abcam, MA, USA) overnight at 4 °C. The HRP-conjugated goat anti-mouse antibodies (1:5000, Santa Cruz, CA, USA) were used as the secondary antibodies. Blots were visualized using Super-Signal West Pico Chemiluminescent Substrate (Pierce, Rockford, IL, USA) and band densities were quantified using Quantity-One software (Bio-Rad, Hercules, CA, USA).

### Enzyme-linked immunosorbent assay (ELISA)

Serum was isolated from the venous peripheral blood from each patient with PV and each healthy control subject and stored at −20 °C. IL-4 and IL-13 protein concentrations in the serum samples were measured using an ELISA kit (R&D, USA). Optical density values were determined at 450 nm using an EL×800 absorbance microplate reader (BioTek, USA).

### T cell *in-vitro* transfections with Nr4a2-overexpressing plasmid and Nr4a2-siRNA

We used an *in-vitro* transfection approach to transfect Nr4a2-overexpressing plasmid (The Biological Company of Shangdong Weizhen) into the CD4+ T cells of six patients with PV. The same method was applied to transfect Nr4a2-siRNA into the CD4+ T cells of six healthy control subjects (The Biological Company of Guangzhou Ruibo).

### Statistical analysis

Student’s *t* test for equality of means was used to compare values. Significance was assumed for *p* < 0.05. All analyses were performed with SPSS software (version 17.0, Chicago, IL, USA).

## References

[1] Zhu H, Chen Y, Zhou Y, Wang Y, Zheng J, Pan M (2012). Cognate Th2-B cell interaction is essential for the autoantibody production in pemphigus vulgaris. J Clin Immunol.

[2] Veldman C, Höhne A, Dieckmann D, Schuler G, Hertl M (2004). Type I regulatory T cells specific for desmoglein 3 are more frequently detected in healthy individuals than in patients with pemphigus vulgaris. J Immunol.

[3] Ujiie H, Shimizu H (2012). Evidence for pathogenicity of autoreactive T cells in autoimmune bullous diseases shown by animal disease models. Exp Dermatol.

[4] Amber KT, Staropoli P, Shiman MI, Elgart GW, Hertl M (2013). Autoreactive T cells in the immune pathogenesis of pemphigus vulgaris. Exp Dermatol.

[5] Nagel A, Lang A, Engel D, Podstawa E, Hunzelmann N, de Pita O, Borradori L, Uter W, Hertl M (2010). Clinical activity of pemphigus vulgaris relates to IgE autoantibodies against desmoglein 3. Clin Immunol.

[6] Brick C, Belgnaoui FZ, Atouf O, Aoussar A, Bennani N, Senouci K, Hassam B, Essakalli M (2007). Pemphigus and HLA in Morocco. Transfus Clin Biol.

[7] Rizzo C, Fotino M, Zhang Y, Chow S, Spizuoco A, Sinha AA (2005). Direct characterization of human T cells in pemphigus vulgaris reveals elevated autoantigen-specific Th2 activity in association with active disease. Clin Exp Dermatol.

[8] Tindemans I, Serafini N, Di Santo JP, Hendriks RW (2014). GATA-3 function in innate and adaptive immunity. Immunity.

[9] Kanhere A, Hertweck A, Bhatia U, Gökmen MR, Perucha E, Jackson I, Lord GM, Jenner RG (2012). T-bet and GATA3 orchestrate Th1 and Th2 differentiation through lineage-specific targeting of distal regulatory elements. Nat Commun.

[10] Ansel KM, Djuretic I, Tanasa B, Rao A (2006). Regulation of Th2 differentiation and Il4 locus accessibility. Annu Rev Immunol.

[11] Satyam A, Khandpur S, Sharma VK, Sharma A (2009). Involvement of TH1/TH2 cytokines in the pathogenesis of autoimmune skin disease—pemphigus vulgaris. Immunol Invest.

[12] Shao DD, Suresh R, Vakil V, Gomer RH, Pilling D (2008). Pivotal Advance: Th-1 cytokines inhibit, and Th-2 cytokines promote fibrocyte differentiation. J Leukoc Biol.

[13] Kidd P (2003). Th1/Th2 balance: the hypothesis, its limitations, and implications for health and disease. Altern Med Rev.

[14] Veldman C, Pahl A, Hertl M (2009). Desmoglein 3-specific T regulatory 1 cells consist of two subpopulations with differential expression of the transcription factor Foxp3. Immunology.

[15] Wang Z, Benoit G, Liu J, Prasad S, Aarnisalo P, Liu X, Xu H, Walker NP, Perlmann T (2003). Structure and function of Nurr1 identifies a class of ligand-independent nuclear receptors. Nature.

[16] Gronemeyer H, Gustafsson JA, Laudet V (2004). Principles for modulation of the nuclear receptor superfamily. Nat Rev Drug Discov.

[17] Saijo K, Winner B, Carson CT, Collier JG, Boyer L, Rosenfeld MG, Gage FH, Glass CK (2009). A Nurr1/CoREST pathway in microglia and astrocytes protects dopaminergic neurons from inflammation-induced death. Cell.

[18] Bandukwala HS, Rao A (2013). 'Nurr'ishing Treg cells: Nr4a transcription factors control Foxp3 expression. Nat Immunol.

[19] Sekiya T, Kashiwagi I, Yoshida R, Fukaya T, Morita R, Kimura A, Ichinose H, Metzger D, Chambon P, Yoshimura A (2013). Nr4a receptors are essential for thymic regulatory T cell development and immune homeostasis. Nat Immunol.

[20] Yokoyama T, Amagai M (2010). Immune dysregulation of pemphigus in humans and mice. J Dermatol.

[21] Ikeda S, Imamura S, Hashimoto I, Morioka S, Sakuma M, Ogawa H (2003). History of the establishment and revision of diagnostic criteria, severity index and therapeutic guidelines for pemphigus in Japan. Arch Dermatol Res.

[22] Takahashi H, Kuwana M, Amagai M (2009). A single helper T cell clone is sufficient to commit polyclonal naive B cells to produce pathogenic IgG in experimental pemphigus vulgaris. J Immunol.

[23] Takahashi H, Amagai M, Nishikawa T, Fujii Y, Kawakami Y, Kuwana M (2008). Novel system evaluating *in vivo* pathogenicity of desmoglein 3-reactive T cell clones using murine pemphigus vulgaris. J Immunol.

[24] Teraki Y, Hotta T, Shiohara T (2001). Skin-homing interleukin-4 and -13-producing cells contribute to bullous pemphigoid: remission of disease is associated with increased frequency ofinterleukin-10-producing cells. J Invest Dermatol.

[25] Wei G, Abraham BJ, Yagi R, Jothi R, Cui K, Sharma S, Narlikar L, Northrup DL, Tang Q, Paul WE, Zhu J, Zhao K (2011). Genome-wide analyses of transcription factor GATA3-mediated gene regulation in distinct T cell types. Immunity.

[26] Peine M, Rausch S, Helmstetter C, Fröhlich A, Hegazy AN, Kühl AA, Grevelding CG, Höfer T, Hartmann S, Löhning M (2013). Stable T-bet(+)GATA-3(+) Th1/Th2 hybrid cells arise *in vivo*, can develop directly from naive precursors, and limit immunopathologic inflammation. PLoS Biol.

[27] Chakir H, Wang H, Lefebvre DE, Webb J, Scott FW (2003). T-bet/GATA-3 ratio as a measure of the Th1/Th2 cytokine profile in mixed cell populations: predominant role of GATA-3. J Immunol Methods.

[28] Caproni M, Giomi B, Cardinali C, Salvatore E, Pestelli E, D'Agata A, Bianchi B, Toto P, Feliciani C, Fabbri P (2001). Further support for a role for Th2-like cytokines in blister formation of pemphigus. Clin Immunol.

